# The Amphibian Chytrid Fungus, *Batrachochytrium dendrobatidis*, in Fully Aquatic Salamanders from Southeastern North America

**DOI:** 10.1371/journal.pone.0044821

**Published:** 2012-09-11

**Authors:** Matthew W. H. Chatfield, Paul Moler, Corinne L. Richards-Zawacki

**Affiliations:** 1 Department of Ecology and Evolutionary Biology, Tulane University, New Orleans, Louisiana, United States of America; 2 Wildlife Research Laboratory, Florida Fish and Wildlife Conservation Commission, Gainesville, Florida, United States of America; Louisiana State University, United States of America

## Abstract

Little is known about the impact that the pathogenic amphibian chytrid fungus, *Batrachochytrium dendrobatidis* (*Bd*), has on fully aquatic salamander species of the eastern United States. As a first step in determining the impacts of *Bd* on these species, we aimed to determine the prevalence of *Bd* in wild populations of fully aquatic salamanders in the genera *Amphiuma*, *Necturus*, *Pseudobranchus*, and *Siren*. We sampled a total of 98 salamanders, representing nine species from sites in Florida, Mississippi, and Louisiana. Overall, infection prevalence was found to be 0.34, with significant differences among genera but no clear geographic pattern. We also found evidence for seasonal variation, but additional sampling throughout the year is needed to clarify this pattern. The high rate of infection discovered in this study is consistent with studies of other amphibians from the southeastern United States. Coupled with previously published data on life histories and population densities, the results presented here suggest that fully aquatic salamanders may be serving as important vectors of *Bd* and the interaction between these species and *Bd* warrants additional research.

## Introduction

The amphibian chytrid fungus, *Batrachochytrium dendrobatidis* (*Bd*), has been associated with amphibian declines and extinctions worldwide [1]–[5]. The severity of impact varies greatly across species, with some species undergoing greater declines (e.g., frogs in the genus *Atelopus* [6]) than others (e.g., proposed carrier species such as *Lithobates catesbeianus* [7] and *L. pipiens* [8]). The impact of *Bd* on many groups, especially semi-aquatic frogs, is well documented; however, the impact of *Bd* on aquatic salamanders is not well understood.

Determining the prevalence of *Bd* in wild populations is a necessary first step in determining the impact that *Bd* has on those populations. Currently, little is known about the prevalence of *Bd* in fully aquatic salamanders. *Bd* has been detected on three species of wild-caught, fully-aquatic salamanders: *Cryptobranchus alleganiensis* [9]–[13], a species which has recently been listed under Appendix III of the Convention on International Trade of Endangered Species (CITES) and a subspecies of which (*C. a. bishop*) has been listed under the U. S. Endangered Species Act; *Andrias japonicus* [14], which is currently listed under Appendix I of CITES; and a single individual *Siren intermedia* from Illinois [15]. In captivity, *Bd* is known from an additional three species: *Amphiuma tridactylum*, *Necturus maculosus* and *Siren lacertina* [16].

Salamanders in the genera *Amphiuma* (3 species), *Pseudobranchus* (2 species), *Siren* (2 species) and *Necturus* (5 species) are fully aquatic and have distributions that are restricted to eastern North America, with the greatest diversity being found along the coastal plain of the southeastern United States. Despite their restricted distributions and their evolutionary and ecological uniqueness, little attention has been given to these groups with respect to *Bd*. In this study, we report the results of a survey identifying the presence and prevalence of *Bd* in salamanders representing all four of these aquatic genera from locations in Florida, Mississippi, and Louisiana.

## Methods

### Ethics Statement

A permit was obtained through the Louisiana Department of Wildlife and Fisheries (permit number LNHP-11-025) for collections made in that state. No permit was obtained for sampling performed in Mississippi and Florida, as those states do not require permits for sampling amphibian species not listed as threatened or endangered. Animal use protocols were approved by Tulane University's Institutional Animal Care and Use Committee (protocol number 0411).

**Figure 1:**
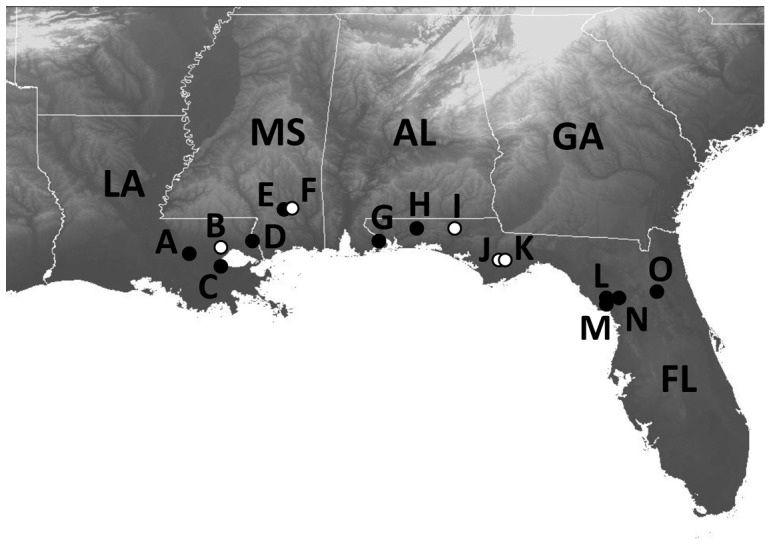
Map of southeastern United States showing collection localities. Filled circles indicate those sites where *Bd* was detected and open circles indicate those sites where *Bd* was not detected. Background shading indicates elevation (lighter  =  higher elevation).

Field surveys for salamanders were conducted at locations in southeastern Louisiana (East Baton Rouge, St. John the Baptist, Tangipahoa and St. Tammany parishes), southern Mississippi (Forrest County) and the panhandle and northern peninsula of Florida (Escambia, Okaloosa, Washington, Liberty, Levy and Putnam counties) (Figure 1). Animals were captured by dipnetting and trapping (baited and unbaited minnow traps placed in suitable habitat overnight). As seasonality has been shown to influence *Bd* prevalence in semi-aquatic species, sampling efforts were focused on the peak prevalence period of March-June [17]–[21].

In order to test for *Bd* infection, salamanders were swabbed by gently rubbing a cotton-tipped swab (MWE113, Advantage Bundling SP, LLC, Durham, NC) 5 times over the dorsum, 5 times on each side, 5 times on the venter, and 5 times on the bottom of each foot. Clean nitrile gloves were used when handling animals, and changed between animals. DNA was extracted from the swabs using the Qiagen DNeasy Blood & Tissue Kit (Qiagen, Inc., Valencia, CA) in a final elution volume of 200 µL, following the protocol for animal tissues.

Quantitative (real-time) PCR assays were used to detect the presence of *Bd* DNA, following Boyle et al. [22]. Preliminary tests showed that PCR inhibition was often present; therefore, all extracted samples were diluted 1∶10 with doubly deionized water prior to use. In addition, 0.7 µL of bovine serum albumin (BSA) was added to each reaction well, as this has been shown to aid in overcoming problems with inhibition [23]. All samples were run in triplicate and scored as positive if at least one replicate tested positive for *Bd*. To confirm that reactions were not inhibited, an internal positive control (VIC_TM_ dye, Applied Biosystems, Inc.) was added to one replicate of each sample. Upper and lower limits of the 95% confidence interval (CI_95_) for *Bd* prevalence were calculated according to Newcombe [24].

## Results

**Figure 2:**
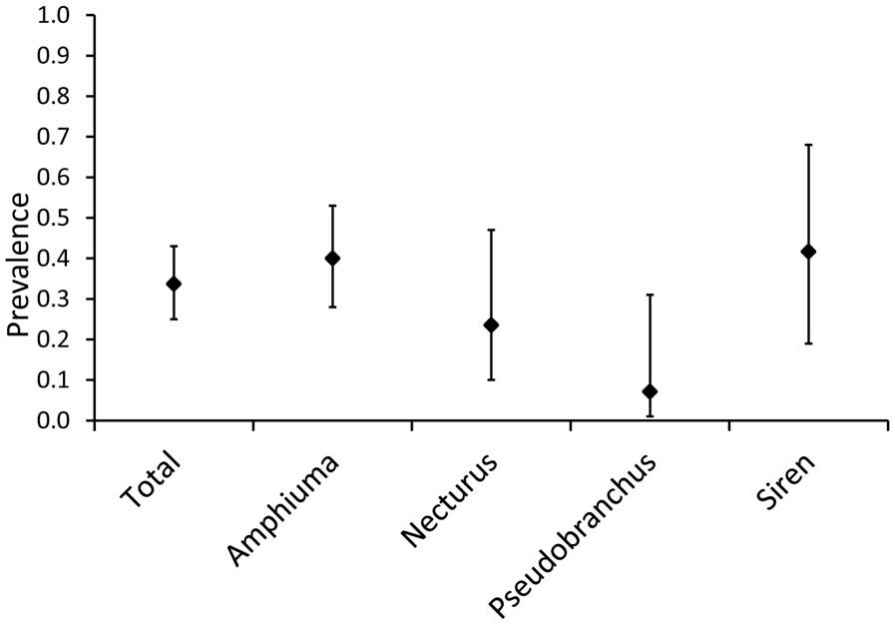
Prevalence of *Bd* infection in all genera combined (Total, N = 98), *Amphiuma* (N = 55), *Necturus* (N = 17), *Pseudobranchus* (N = 14), and *Siren* (N = 12). Bars indicate 95% confidence interval.

**Figure 3:**
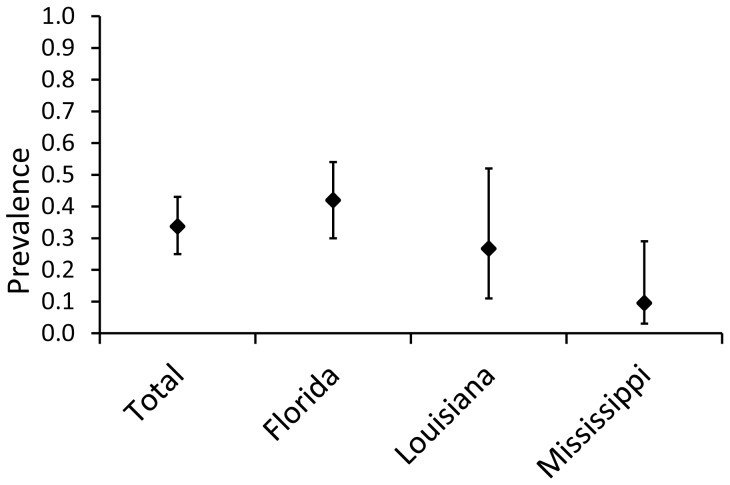
Prevalence of *Bd* infection in all sampling localities combined (Total, N = 98), Florida (N = 62), Louisiana (N = 15), and Mississippi (N = 21). Bars indicate 95% confidence interval.

**Figure 4:**
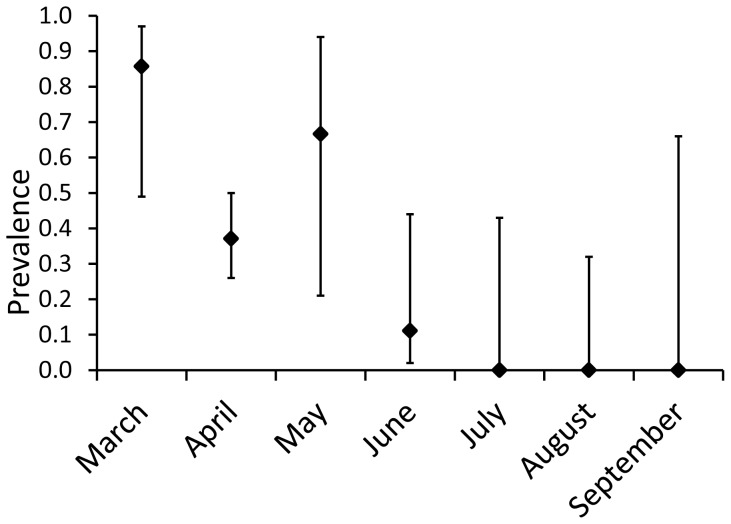
Prevalence of *Bd* infection for the six months in which sampling was heaviest: March (N = 7), April (N = 62), May (N = 3), June (N = 9), July (N = 5), and August (N = 8). Bars indicate 95% confidence interval.

The prevalence of *Bd* among all salamanders surveyed in this study was 0.34 (CI_95_ = 0.25–0.43, Table 1), although prevalence differed among genera (Fisher's Exact Test: P = 0.009, Figure 2). *Siren* (N = 12, prevalence  = 0.42, CI_95_ = 0.19–0.68) and *Amphiuma* (N = 55, prevalence  = 0.40, CI_95_ = 0.28–0.53) had the highest *Bd* prevalence, followed by *Necturus* (N = 17, prevalence  = 0.24, CI_95_ = 0.10–0.47) and *Pseudobranchus* (N = 14, prevalence  = 0.07, CI_95_ = 0.02–0.31). Infections did not show any clear geographic pattern and *Bd* prevalence did not differ among states (Fisher's Exact Test: P = 0.51, Figure 3). Out of 98 individuals tested, 94 were captured from March through August (Figure 4). *Bd* prevalence differed across these six months with March (N = 7, prevalence  = 0.86, CI_95_ = 0.49–0.97), April (N = 62, prevalence = 0.37, CI_95_ = 0.26–0.50) and May (N = 3, prevalence  = 0.67, CI_95_ = 0.21–0.94) having higher prevalence than June (N = 9, prevalence = 0.11, CI_95_ = 0.02–0.44), July (N = 5, prevalence = 0, CI_95_ = 0–0.43) or August (N = 8, prevalence = 0, CI_95_ = 0–0.32).

**Table 1:** Results of survey for *Batrachochytrium dendrobatidis* in fully aquatic salamanders from Florida, Louisiana, and Mississippi.

Species	Site*	Lat/Long	State	N	# Positive	Prev.
*Amphiuma means*	E	31.154/−89.245	MS	4	1	0.25
*A. means*	F	31.113/−89.142	MS	14	1	0.07
*A. means*	N	29.326/−82.772	FL	4	3	0.75
*A. means*	L	29.516/−82.876	FL	8	8	1
*A. means*	M	29.286/−82.845	FL	7	4	0.57
*A. pholeter*	G	30.516/−87.322	FL	1	0	0
*A. tridactylum*	A	30.364/−91.121	LA	11	2	0.18
*Amphiuma* sp.	I	30.789/−85.756	FL	2	0	0
*Amphiuma* sp.	L	29.516/−82.876	FL	4	4	1
Total *Amphiuma*				55	23	0.4
*Necturus alabamensis*	J	30.248/−85.005	FL	3	0	0
*N. alabamensis*	K	30.259/−84.972	FL	2	0	0
*N. alabamensis*	H	30.700/−86.576	FL	10	3	0.3
*N. beyeri*	D	30.539/−89.875	LA	2	1	0.5
Total *Necturus*				17	4	0.24
*Pseudobranchus axanthus*	O	29.542/−81.837	FL	13	1	0.08
*P. striatus*	I	30.789/−85.756	FL	1	0	0
Total *Pseudobranchus*				14	1	0.07
*Siren intermedia*	B	30.397/−90.429	LA	1	0	0
*S. intermedia*	C	30.108/−90.435	LA	1	1	1
*S. intermedia*	E	31.154/−89.245	MS	1	1	1
*S. intermedia*	F	31.113/−89.142	MS	2	0	0
*S. lacertina*	O	29.542/−81.837	FL	3	0	0
*S. lacertina*	L	29.516/−82.876	FL	3	2	0.67
*Siren* sp.	G	30.516/−87.322	FL	1	1	1
Total *Siren*				12	5	0.42
Total Salamanders				98	33	0.34

* Sites correspond to those given in Figure 1.

## Discussion

Prevalence of *Bd* infection in the fully aquatic salamander species was high (0.34 with a CI_95_ of 0.25–0.43) during the period sampled in this study. The peak infection period observed in this study (March–May) corresponds well to the peak infection period observed in other (semi-aquatic) species in this region [17]–[21]. The sharp decline in prevalence in June may indicate the start of a season of low prevalence that is also seen in semi-aquatic species in this region [21]. It is important to note, however, that sampling in July and August was limited to 13 individuals collected from one site in Mississippi. Additional sampling is needed to fully address the issue of seasonality.

Alternatively, the greater thermal stability of aquatic environments suggests that fully aquatic amphibian species might be year-round hosts of *Bd*, serving as a reservoir for the pathogen that could infect semi-aquatic species during their breeding season. The preferred environmental temperatures of two species of fully aquatic salamanders, *Cryptobranchus alleganiensis* and *Necturus maculosus* (11.6–21.7°C and 9.1–20.2°C, respectively, [25] overlap with the lower end of the optimal growth range of *Bd* (17–25°C [26]–[29]). The preferred temperature of a third species, *Amphiuma tridactylum*, is marginally higher (26.3°C [30]), although still within the range where *Bd* can survive [28]. Species of *Amphiuma* also rely heavily on crayfish burrows as retreat sites [30]. These microhabitats provide suitable environments for *Bd* growth (18–20°C [30]) and may, in conjunction with aestivating *Amphiuma*, be acting as reservoirs for *Bd* during warm summer months in subtropical climates and, thus, may contribute to the high prevalence of *Bd* (0.40) found in *Amphiuma* in this study. Lastly, *Siren* and *Pseudobranchus* are able to survive in seasonal wetlands or periods of drought by burrowing into the substrate and forming a desiccation-resistant cocoon [31]–[33]. As with *Amphiuma*, aestivating *Siren* and *Pseudobranchus* may be acting as *Bd* reservoirs. Additional research is needed to elucidate the role that these complex adaptations play in *Bd*-host interactions.

We did not detect any geographic patterns with respect to the presence or prevalence of *Bd* infection. Positive individuals were detected in all three of the states sampled and prevalence was not significantly different across states. Importantly, all of our sites were of similar latitude (29.5–31.2° N) and likely experienced similar climates. Other studies conducted in the southeastern United States support the widespread distribution of *Bd* in this region and have found similarly high infection prevalences in other amphibian species during certain times of the year. For example, Gaertner et al. [18] found an infection prevalence of 0.83 in cricket frogs (*Acris crepitans*) sampled in May from a site in central Texas. In Virginia, Pullen et al. [19] found a peak prevalence of 0.45 across 13 semi-aquatic frog and salamander species. Sampling across seasons, Rothermel et al. [17] found a prevalence of 0.18 in 12 species across four sites in Georgia, North Carolina, and South Carolina.

The high prevalence of *Bd* found in this study is significant and suggests that additional studies are needed to understand the impact of *Bd* on these taxa. The absence of *Bd*-related die-offs of amphibians in eastern North America suggests that *Bd* is acting in an endemic fashion in the region. Currently, little is known about the effects of *Bd* on amphibian populations in the absence of mass die-offs; however, previous studies have demonstrated a negative impact of *Bd* on semi-aquatic frog species following historic mass die-offs [34] and altered anti-predator defense strategies in *Bd*-infected versus uninfected tadpoles (e.g., [35]). The Ozark Hellbender (*Cryptobranchus alleganiensis bishopi*), another fully aquatic salamander from the eastern United States, has recently been listed as federally endangered and also has a high reported prevalence of *Bd* in the wild (0.33) [13]. While it is difficult to ascertain the role that *Bd* has played in the decline of that species, it seems likely that many stressors acting in concert may be reducing survival and reproduction (e.g., [12], [36]). Given the presence of *Bd* infection in fully aquatic salamanders, the threat that *Bd* poses will ultimately depend upon the extent to which these groups exhibit symptoms of chytridiomycosis, the disease caused by *Bd*, or other subclinical effects. Some terrestrial and semi-aquatic salamander species are known to harbor antifungal bacteria and compounds on their skin [37]–[39]. While many fully aquatic salamander species are commonly known to have abundant mucus secretions, research is needed to identify whether these secretions contain antifungal compounds active against *Bd*.

Lastly, the extraordinarily high densities reached by many species of fully aquatic salamanders in the wild suggest that these species may play a disproportionately large role in *Bd*-amphibian interactions. For example, populations of *Siren lacertina* and *Amphiuma means* in northern Florida have been found to reach densities of 1.3 and 0.28 salamanders/m^2^, respectively [40]; and populations of *Siren intermedia* in Texas and Missouri have densities of 0.33–1.1 and 1.35–2.17 salamanders/m^2^, respectively [41]–[43]. Furthermore, many of the species examined in this study are large, making them significant contributors to biomass in southeastern aquatic ecosystems. For example, estimates of the standing crop biomass of *S. lacertina* is 233 g/m^2^, *A. means* is 44 g/m^2^, and *S. intermedia* is 9.66–72.2 g/m^2^ [40]–[43]. The high densities at which these salamanders occur in nature suggests that fully aquatic salamanders of the eastern United States are ecologically important. Therefore, understanding potential threats to these species is important in the conservation and management of aquatic ecosystems in this region. Additionally, these species may be harboring large amounts of *Bd*, thus contributing disproportionately as vectors to *Bd*-host interactions involving other (semi-aquatic) amphibian groups.
