# Correction: The Amphibian Chytrid Fungus, *Batrachochytrium dendrobatidis*, in Fully Aquatic Salamanders from Southeastern North America

**DOI:** 10.1371/journal.pone.0271363

**Published:** 2022-07-08

**Authors:** Matthew W. H. Chatfield, Paul Moler, Corinne L. Richards-Zawacki

The following text in the Ethics Statement is incorrect: No permit was obtained for sampling performed in Mississippi…as [this state] does not require permits for sampling amphibian species not listed as threatened or endangered.

Instead, the Ethics Statement of this article [1] has been updated to include the following information: Scientific collecting permits were obtained from the Mississippi Department of Wildlife, Fisheries and Parks (permit numbers 0127113 and 0428111) for collections made in Mississippi.

These permits covered the sampling of amphibians which took place in Spring 2011, and allowed for the capture and release of animals, as described in this article [1]. One of the permits (0127113) was obtained after the sampling was carried out, and was retroactive.
